# How do policy measures affect the ability of poverty-stricken households to cope with external shocks? From the perspective of differences in the human capital endowment of households

**DOI:** 10.3389/fpubh.2024.1361303

**Published:** 2024-10-31

**Authors:** Xinyue Chang, Qianqian Zhang, Tao Li

**Affiliations:** ^1^College of Geographical Sciences, Southwest University, Chongqing, China; ^2^Department of Tourism Management, Jinzhong University, Shanxi, China

**Keywords:** human capital, external shocks, COVID-19, policy measures, poverty-stricken households

## Abstract

Human capital plays a vital role in poverty-stricken households' efforts to cope with external shocks. Improving the human capital of poverty-stricken households to help them address external shocks can enhance the sustainability of poverty-stricken household livelihoods and support poverty alleviation. In this study, households with dependent children and older members were selected from questionnaires covering 6,463 poverty-stricken households from 33 poverty alleviation districts and counties in Chongqing municipality, China. Multiple linear regression model and stepwise regression methods were then used to compare the effects of the number of household members supported and the number of those working on the increase in income among poverty-stricken and nonpoverty-stricken households. Finally, the correlations between policy measures, dependent household members and household labor were examined. The results show the following: (1) Policy measures can alleviate the negative impact of a household's dependent population on household income. (2) Poverty-stricken households' access to policy support is closely related to the characteristics of their human capital. The household's dependent members and laborers are effectively protected by safety net and cargo net policies. (3) Policy measures can mitigate the impact of COVID-19 on the income of nonagricultural laborers while increasing the income of agricultural laborers. The findings show that the ability of poverty-stricken households to overcome poverty and deal with external shocks can be increased by revitalizing rural industries, linking the development of rural industries with the income of poverty-stricken households, and rationally using rural labor resources.

## 1 Introduction

From 2020 to 2023, the COVID-19 pandemic had adverse effects on human development. Data issued by the World Health Organization (WHO) revealed that 768,983,095 cases of COVID-19 had been confirmed worldwide, including 6,953,743 deaths as of August 2, 2023, at 16:50 p.m. CET. Moreover, on December 21, 2021, the World Bank (WB) published an article stating that the COVID-19 pandemic caused the sharpest decline in income among the poorest 40% of the population. The decrease in income forced ~100 million people into extreme poverty. Several studies have demonstrated that relying solely on the power of poverty-stricken households made it difficult to cope with the adverse effects of the COVID-19 pandemic (1, 2, 4). Therefore, an increasing number of scholars, governments and international organizations have called for more policy measures to mitigate the adverse effects of the COVID-19 pandemic on poor populations (5–7).

The effects of policy measures to mitigate the adverse effects of external shocks are manifested in two ways. First, policy measures act as safety nets to buffer the adverse effects of external shocks. For example, assistance to poverty-stricken households in the form of medical insurance (8, 9), unemployment insurance (10), and assistance in kind and funds (11, 12). Safety nets mitigate the adverse impacts of external shocks on poverty-stricken households from the perspective of external risk defense, which is suitable for mitigating the short-term adverse impacts of external shocks on poverty-stricken households while having a relatively small effect on the long-term self-development ability of poverty-stricken households to cope with external shocks (13, 14). In addition, safety nets enhance the long term self-development ability of poverty-stricken households through indirect means, such as by influencing labor redistribution (15) and capital-in-distribution strategies (16). Second, policy measures act as cargo nets to increase poverty-stricken households' ability to escape the poverty trap caused by external shocks. For example, infrastructure can be improved to provide good production conditions for poverty-stricken households (17) and enhance the employment ability of poverty-stricken laborers through skills training (18). These policy measures can enhance the long-term self-development ability of poverty-stricken households to cope with external shocks, which contributes to the continuous improvement of their living standards. However, many papers have reported the effectiveness of policy measures in cushioning the short-term adverse effects of external shocks (12, 19, 20), but few have reported that effective policy measures successfully improve the long-term ability of poverty-stricken households to self-develop. Thus, it is necessary to find an effective way to increase the long-term ability of poverty-stricken households to cope with external shocks.

The ability of poverty-stricken households to cope with external shocks is closely related to their human capital. Human capital represents the skills, knowledge, ability to work and good health that, together, enable people to pursue different livelihood strategies using different forms of assets and attain their livelihood goals (21, 22). The fundamental constraint on improving poverty-stricken households' livelihood level is insufficient human capital (23). Thus, enhancing poverty-stricken households' human capital is crucial to their long-term ability to cope with external shocks. However, several problems exist with enhancing the human capital of poverty-stricken households through policy measures. First, how can the human capital endowment of poverty-stricken households be measured? The human capital of poverty-stricken households can be measured from two aspects: quality and quantity. However, compared with the quantity of human capital, measuring the quality of human capital is somewhat subjective from aspects such as education (24), skills (25) and workforce capacity (26). In fact, human capital endowment and household population composition are closely related, and they are more accurately measured in terms of household population composition. Therefore, household population composition must be considered when developing policies to provide security for poverty-stricken households most vulnerable to external shocks (27). Second, what is the role of human capital in poverty-stricken households affected by external shocks? External shocks are a significant factor in the vulnerability of poverty-stricken households to poverty (29). Human capital is not only an important reason for rural households falling into poverty traps but also plays a major role in household agricultural production and nonagricultural employment. Improving the human capital level of poverty-stricken households is crucial for enhancing the ability of poverty-stricken household laborers to cope with external shocks. Although human capital helps poverty-stricken households cope with risk shocks (30, 31), it is not clear how to directly or indirectly enhance human capital. Neither is it clear how human capital is directly or indirectly related to access to policy measures. Thus, clarifying the relationship between the acquisition of policy measures and human capital is important. Third, what are the respective effects of policy measures on poverty-stricken households with different types of human capital? Some research studies have focused on how a single policy has enabled poverty-stricken households to cope with external shocks (32, 33); however, they have neglected the systemic nature of China's poverty alleviation measures. Policy measures do not exist in isolation but are constructed as organic systems containing safety and cargo nets (34). Hence, attention should be given to the multiple impacts of different policy measures on rural households for coping with external shocks. In other words, practical measures are required to enhance the ability of poverty-stricken households to cope with external shocks.

Targeted poverty alleviation in China is an excellent case for exploring these problems. To solve poverty caused by external shocks, the Chinese government has implemented various policy measures, including those for industry, employment, health, and other aspects, to ensure the livelihood of poverty-stricken households. The majority of studies have focused on risk perceptions (35, 36) and the prevention of risk in poverty-stricken households (37). Some scholars have noted the role of training participation (38), social networks (39), agricultural insurance (40), and livestock insurance (41) in the risk impact on poverty-stricken households. However, little attention has been given to the role of policy measures in supporting the response of poverty-stricken households' human capital to external shocks. Therefore, the effects of policy measures on the ability of poverty-stricken households to cope with external shocks should be thoroughly explored.

To fill these research gaps, we collected questionnaire data from 6,463 households in national-level, contiguous poverty-stricken areas in China to explore how local policy measures affect the ability of poverty-stricken households to cope with external shocks. The paper is structured as follows: Section 2 presents the theoretical analysis. We then introduce the research area and data in Section 3. Section 4 presents the econometric results and discussion. Finally, we conclude this study and discuss the implications of our findings.

## 2 Theoretical analysis

### 2.1 Targeted poverty alleviation policy measures in China

**Figure 1:**
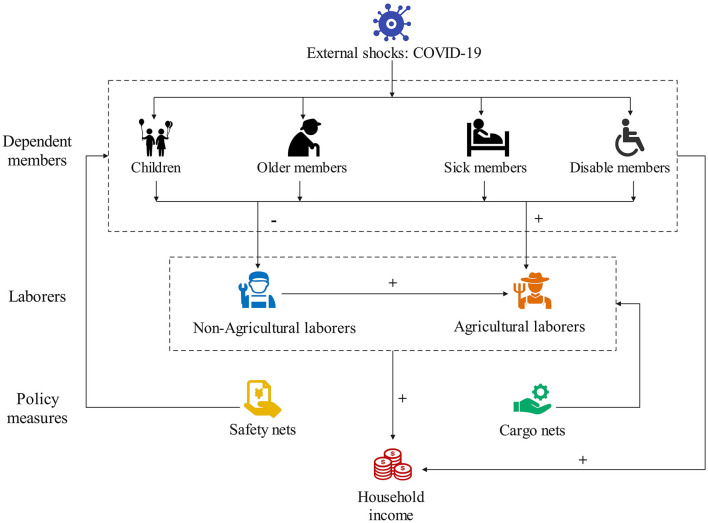
The mechanism of policy measures in the response of poverty-stricken households to external shocks.

Since 2013, the Chinese government has implemented targeted poverty alleviation (TPA), which takes antipoverty control as a major political goal and livelihood project to construct a prosperous society before 2020 (42). TPA is a special mechanism that accurately allocates poverty alleviation resources to poor populations through institutional arrangements and policy support (43). The policy measures for alleviating poverty for poverty-stricken households can be divided into two types: safety nets and cargo nets (34) (Figure 1).

Safety net policies provide basic livelihood protection for poverty-stricken households that suffer from external shocks. Under such policies, the government provides direct economic and material assistance to prevent poverty-stricken households from falling into poverty traps. However, the effectiveness of safety net policies is short-term. Safety net policies cannot stimulate the endogenous dynamics of poverty-stricken households in the long term. Safety net policies mainly include urban and rural medical insurance, temporary medical assistance, education subsidies, subsistence allowances (Dibao), disability awards, social old-age insurance and funds to renovate rural dilapidated houses, which support poverty-stricken households when they suffer from external shocks. External shocks often lead to a loss of labor days or lowered productivity for affected household members and their caregivers, which directly reduces their household income. For example, Dibao aims to address poverty by providing cash transfers to people in households below an income threshold. Families whose household per capita income falls below the local Dibao assistance line can apply for and receive monthly Dibao benefits, lifting their income level to the local Dibao threshold level. Additionally, the government has renovated dilapidated rural houses to help poverty-stricken households with the worst housing conditions solve the most basic housing safety problems Janvry. Safety net policies are critically important to prevent households transitioning out of poverty from falling back into chronic poverty.

In contrast, cargo net policies provide poverty-stricken households with the ability to escape poverty based on their livelihood capital endowments. Such policies have a long-term impact on poverty reduction through providing targeted policy assistance (44, 45). Cargo net policies include public welfare jobs, land transfers, industry shares, vocational training, relocation and microcredits, which play a comprehensive role in supporting poverty-stricken households on the edge of poverty. Public welfare jobs, such as those related to environmental sanitation and forest protection, are provided to the poor population as a compensation scheme offering additional job opportunities. Poverty-stricken households that accept these jobs can obtain wages and broaden their income channels, thus increasing the overall income of their families. In addition, land transfers, industry shares, vocational training, relocation and microcredits enhance the human capital of poverty-stricken households and establish paths out of chronic poverty. Under relocation policies, poverty-stricken households living villages with insufficient carrying capacity, infrastructure and public services or in disaster-affected areas can move to safer, better-provisioned areas (46). In other words, cargo net policies aim to help people escape chronic poverty, which is crucial for poverty-stricken households suffering from external shocks.

### 2.2 The adverse effects of external shocks on poverty-stricken households' human capital

**Figure 2:**
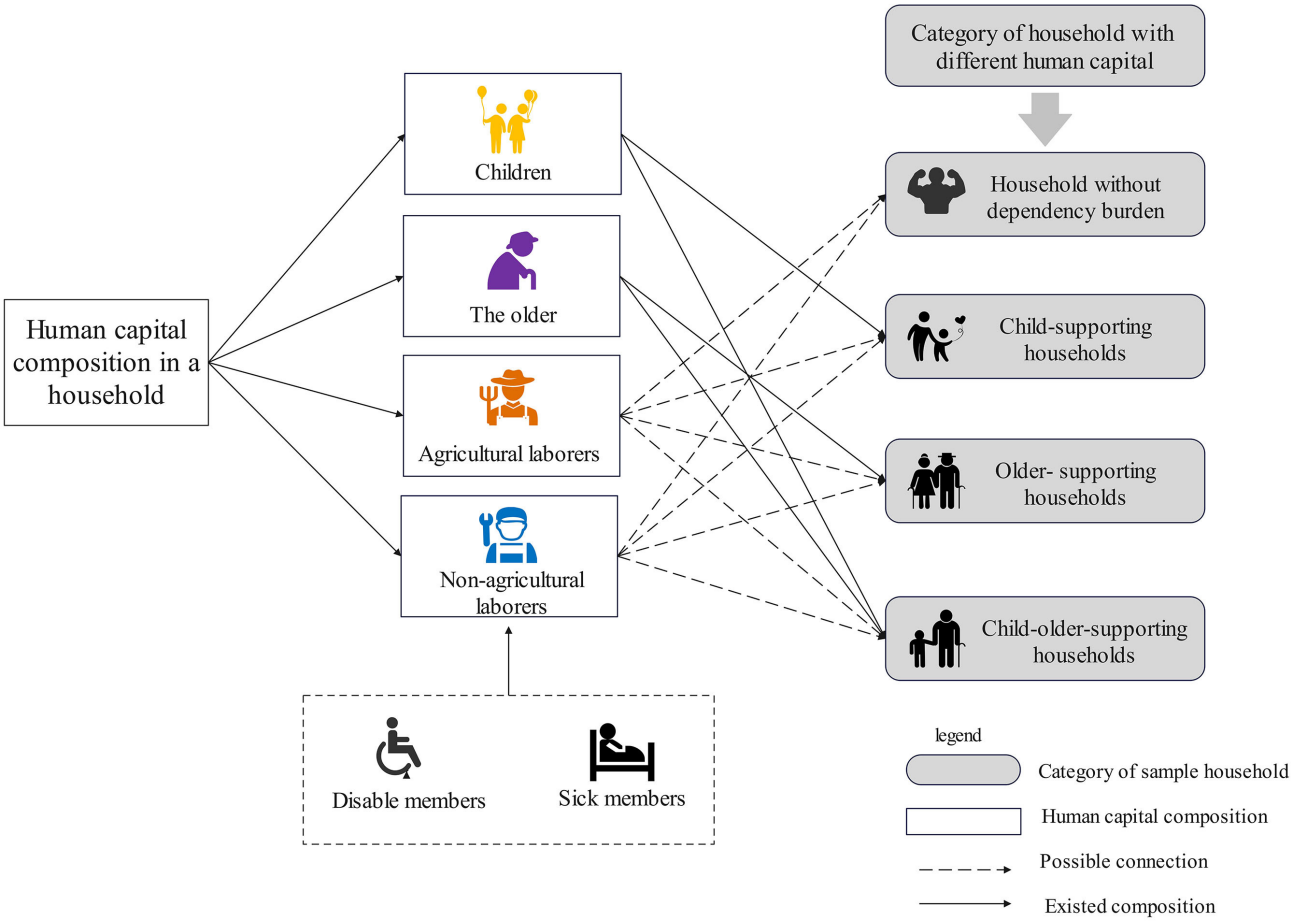
The category of household with different human capital.

In the stable phase, a household's human capital comprises children, adults and older individuals. Among these, children and older individuals are the main dependent members. Additionally, sick and disabled members caused by external shocks also belong to dependent members (Figure 2). According to the “dependency burden hypothesis,” dependent family members are not part of the economically active population. They are consumers of social material wealth and do not participate in the labor market. According to this definition, the dependency of children and older individuals is different. In rural China, children are generally fully dependent, spending time in school and creating little economic value for their families. Older people with better health will continuously take part in agricultural production and provide as much economic value for their families as possible (47). The impact of dependent members on poverty-stricken household income is reflected in two aspects. First, dependent members increase the consumption expenditures of poverty-stricken households. Dependent members increase household expenditures on nutrition, school fees and medical expenses, increasing the household financial burden (48). Second, dependent members affect the allocation of human capital. Specifically, to care for the dependent members in a poverty-stricken household, laborers must relinquish high-paying opportunities far from their hometowns to work closer to their dependent household members to better care for them. Moreover, caring for dependent members often leads to a loss of work days or less productivity for household laborers (49), further reducing poverty-stricken household agricultural and nonagricultural income. Thus, to sustain household livelihood, children may shorten their years of schooling, and older people may expand their years of work (50), potentially hindering the long-run improvement of the quality of poverty-stricken household human capital.

The adverse effects of external shocks on poverty-stricken households' human capital follow the two aspects mentioned above. For example, the COVID-19 pandemic directly affects the health status of members of poverty-stricken households, increasing the number of dependent members and decreasing the number of laborers in families. The data released by the Chinese Academy of Social Sciences (CASS) show that more than 76.8% of surveyed rural households will have their annual income reduced by 5% or more (51). In addition, due to the impact of COVID-19, the Brookings Institute estimates that the number of people living in extreme poverty worldwide will increase by ~40 million compared with that in 2019. On the other hand, to reduce the risk of COVID-19 pandemic infection, many enterprises have shut down in urban regions, especially in labor-intensive industries, such as catering, accommodation, retail, logistics and transportation. This has led to shortened employment periods and job loss for laborers, forcing rural migrants to return to agricultural production. Moreover, in rural regions, pandemic control measures disrupted logistics distribution, which made transporting and unmarketability of agricultural products difficult. Moreover, owing to the lockdown of villages and the strict control of intercity traffic, spring plow preparation and agricultural production in rural areas were affected, impacting the agricultural income of agricultural laborers (52). All of these factors adversely affect the effectiveness of human capital in improving household income.

### 2.3 The connection between TPA policy measures and human capital

To alleviate the economic burden of poverty-stricken households, the government has built powerful safety nets to support their dependent members. For sick individuals, the government has reduced the pressure of medical expenses for poverty-stricken households by introducing urban and rural medical insurance (53), disease insurance measures and temporary medical assistance. For the education expenses of children, the government has implemented tuition fee reduction, free nutritious meals and education subsidies for poverty-stricken students along with compulsory education. For disabled and older persons, disability awards, subsistence allowances (Dibao) and social old-age insurance (54) are provided to ensure their basic livelihood. These safety net policies can effectively mitigate the adverse impacts of the dependent population on the income of poverty-stricken households in the case of external shocks.

To improve the quality of poverty-stricken households' human capital, the government has implemented various cargo net policy measures. Specifically, new rural business entities, such as village collective industries and cooperatives, are cultivated to absorb poverty-stricken households' livelihood capital, such as cropland and agricultural laborers (55, 56). Moreover, effective connections between collective industrial benefits and the individual interests of poverty-stricken households enhance the economic output of agricultural laborers. For example, public welfare jobs (57), vocational training and microcredit are used to support poverty-stricken households in increasing agricultural income (58). Following the COVID-19 pandemic, nonagricultural laborers in poverty-stricken households began to engage in agricultural production. An increase in the number of agricultural laborers can increase the efficiency of agricultural output. The increase in agricultural income has also helped mitigate the loss of migrant income caused by the impact of COVID-19.

## 3 Data sources and research methods

### 3.1 Data sources

#### 3.1.1 Study area and data

Chongqing municipality is located in Southwest China, in the upper reaches of the Yangtze River and the hinterland of the Three Gorges Reservoir area. The city covers an area of 82,300 km^2^ and has a complex geomorphological structure and diverse topography. Mountains and hills account for 70% of the total area. The Qinba Mountains and the Wuling Mountains are national-level contiguous areas in extreme poverty and are distributed in northeast Chongqing and southeast Chongqing, respectively. Among the 38 districts and counties under Chongqing's jurisdiction, 33 involved in poverty alleviation were selected. They were divided into three types to implement TPA policy measures according to their poverty status: national-level supporting counties, municipality-level supporting counties and general supporting counties. The number of municipalities that correspond to the three types are 14, 4, and 15, respectively. In 2014, 1.659 million registered poor people were living in Chongqing municipality (59). Chongqing successfully supported the rural population below the current poverty line exit poverty by the end of 2020, achieving an incidence rate of poverty of 0.13%.

However, the impact of the COVID-19 pandemic posed a new threat to the livelihoods of poverty-stricken households. Rural areas had limited epidemic defense capabilities and mechanisms, and poverty-stricken households had little awareness of the epidemic and adopted insufficient self-protection measures during the early stages, seriously impacting productive life. However, the government has invested a large amount of resources in the study area, including providing diversified subsidies, stabilizing employment, and enacting policies to benefit agriculture. For example, the government has issued 13 policy measures to stabilize employment and 10 policy measures to alleviate industrial poverty, aiming to ensure that agricultural and nonagricultural laborers can earn a steady income. The livelihoods of poverty-stricken households in the study area are affected by both external shocks and policy measures, providing an opportunity to explore the role played by policy measures in the ability of poverty-stricken households to cope with external shocks.

The data used in this paper were collected from a questionnaire of poverty-stricken households in 33 counties in Chongqing municipality, China, from November to December 2020. The questionnaire included demographic characteristics, household income, support from policy measures and the impact of COVID-19 on poverty-stricken households. The poverty-stricken household survey was conducted in three steps (60). The data used in this paper were collected from a questionnaire of poverty-stricken households in 33 counties in Chongqing municipality, China, from November to December 2020. The questionnaire included demographic characteristics, household income, support from policy measures and the impact of COVID-19 on poverty-stricken households. The poverty-stricken household survey was conducted in three steps (61). The respondents were householders, and other household members could participate as supplements. Each rural household interview lasted for ~1–2 h. Of the 7,000 questionnaires that were distributed, a total of 6,463 valid questionnaires were obtained after excluding outliers and irrational questionnaire responses, for an effective response rate of 92.33%.

#### 3.1.2 Sample description

In the sample, male household heads constituted ~81.76% of the sample household respondents, and 88.24% of the household heads were of Han nationality. The educational level of household heads was generally low, with nearly 79.67% of household heads having an education level of primary school or below. More than 78% of the household heads were over 50 years old, revealing the common characteristic of population aging. In the sample, 63.89% of the households were poverty-stricken, and 36.11% were nonpoverty-stricken. Regarding household size, poverty-stricken households with 0–3 persons and 4–6 persons accounted for 56.52% and 39.78%, respectively, of the sample households. Household income was concentrated in the categories 0–18,000 CNY and 18,000–36,000 CNY, accounting for 23.91% and 24.90%, respectively, of the sample households (Table 1). Based on the human capital composition of a household, poverty-stricken households were classified into four categories: households without dependency burdens (0 older members, 0 children), child-supporting households (0 older members, number of children >1), older-supporting households (0 children, number of older members >1) and children–older-supporting households (number of children >1, number of older members >1). In the sample, the largest number of households with dependency burdens were children-supporting households and older-supporting households, whose proportions were 15.40 and 41.81%, respectively.

**Table 1:** Basic information on the sample poverty-stricken households (sample size = 6,463).

**Variable**		**Sample (household)**	**Percentage (%)**	**Variable**		**Sample (household)**	**Percentage (%)**
Gender of the householder	Male	5,284	81.76	Type of the household	Poverty-stricken household	4,129	63.89
	Female	1,179	18.24		Nonpoverty-stricken households	2,334	36.11
Nationality of the householder	Han	5,703	88.24	Household burden	No dependency burden	1,919	29.69
	Minority	760	11.76		Child-supporting	995	15.40
Educational level of the householder	Primary school and below	5,149	79.67		Older-supporting	2,702	41.81
	Middle school	1,168	18.07		Child-supporting and older-supporting	847	13.11
	High school, vocational school	118	1.83	Household size (persons)	(0, 3]	3,653	56.52
	College degree or above	28	0.43		[4, 6]	2,571	39.78
Age of the householder	(0, 40)	223	3.45		[7, +∞)	239	3.70
	[40, 50)	1,131	17.50	Household income (CNY)	[0, 18,000)	1,545	23.91
	[50, 60)	1,978	30.60		[18,000, 36,000)	1,609	24.90
	[60, 70)	1,603	24.80		[36,000, 50,000)	1,168	18.07
	[70, +∞)	1,528	23.64		[50,000, 70,000)	1,113	17.22
					[70,000, +∞)	1,028	15.91

### 3.2 Research methodology

#### 3.2.1 Variable selection

Rural household income, characteristics of rural household human capital, policy measures, and household capital were selected as variables based on the theoretical analysis.

Dependent variable: The most basic indicator of poverty status is rural household income. It reflects households' overall welfare level and is the most direct and effective means to cope with risks (7). When poverty-stricken households suffer from COVID-19 shocks, agricultural production efficiency decreases, and the number of nonagricultural laborers decreases, which affects household income. The variable “change in household income after the COVID-19 pandemic” is used to measure the impact of the COVID-19 pandemic on the household income of poverty-stricken households.

Core variable: Each individual in the household experiences childhood, adulthood, or old age. Parental nurturing and education support the accumulation of human capital in childhood. In adulthood, people work to earn income for personal consumption, savings, and the support of parents and children. When people reach old age, their working ability declines, and they depend on pension insurance, child support and savings obtained during their working years for consumption (62). The number of dependent family members (63) and laborers (64) in a rural household impacts the household's income. Specifically, the “number of older members” and “number of children” describe the impact of dependent family members on the income of poverty-stricken households, whereas the “number of nonagricultural laborers” and “number of agricultural laborers” describe the impact of household laborers on the income of poverty-stricken households.

The control variables, namely, household size (65), household livelihood capital (37), policy measures (66, 67) and household risk factors (68, 69), affect household income. Among them, the “household size” variable is the number of household members; “total household income” reflects household livelihood capital; “government subsidies” indicates the situation of support policies; and the “hospitalization or not” of rural household members in 2020 indicates household risk factors. The definitions and descriptive statistical characteristics of each variable are shown in Table 2.

**Table 2:** Definitions and summary statistics of the variables.

**Variable**	**Definitions**	**Mean**	**SD**	**Value range**
**Dependent variables**				
Changes in household income after COVID-19	The increase or decrease in household income of poverty-stricken households after the epidemic: A sharp drop in income = 0 A slight drop in income = 1 Basically unchanged income = 2; income increase = 3	2.59	0.85	0–4
**Core variables**				
Number of older members	The number of older members in a rural household	0.79	0.82	0–6
Number of children	The number of children in a rural household	0.39	0.69	0–5
Number of nonagricultural laborers	The number of non-agricultural laborers in a rural household	0.83	0.97	0–6
Number of agricultural laborers	The number of agricultural laborers in a rural household	1.09	0.87	0–8
**Control variables**				
Household size	Total population of poverty-stricken households	3.31	1.72	0–13
Disabled	The number of disabled members in a rural household	0.71	0.75	0–5
Hospitalization	Whether poverty-stricken household members are hospitalized; no = 0; yes = 1	0.11	0.31	0–1
Government subsidies	Ln(*x*); *x* = Income from policy assistance projects obtained by poverty-stricken households	7.14	3.21	0–13.08
Household income	Ln(*x*); *x* = Total household income of poverty-stricken households	10.37	0.90	0–13.80
**Policy variables**				
Subsistence allowances (SA)	Whether a rural household obtains an SA: no = 0; yes = 1	0.22	0.41	0–1
Renovation of rural dilapidated houses (RH)	Whether a rural household obtains an RH: no=0; yes=1	0.27	0.45	0–1
Public welfare jobs (WJ)	The number of public welfare jobs in a rural household	0.21	0.44	0–3
Relocation (RL)	Whether a rural household obtains RL: no = 0; yes = 1	0.04	0.18	0–1
Industry share (IS)	Whether a rural household obtains IS: no = 0; yes = 1	0.13	0.34	0–1
Land transfer (LT)	Ln(*x*); *x* = income came from land transfer	1.75	2.59	0–11.3504
Microcredit (CR)	Whether a rural household obtains CR: no = 0; yes = 1	0.30	0.46	0–1

#### 3.2.2 Model setting

To estimate the effects of policy measures on the response of poverty-stricken households to the COVID-19 shock, we use a multiple linear regression model. The first objective is to compare the effect of the number of children on the increase in income between poverty-stricken households with children and their nonpoverty-stricken counterparts. Second, we compare the effect of the number of older members on the increase in income between poverty-stricken households with older members and their nonpoverty-stricken counterparts. Finally, we measure the role of policy measures by analyzing the impact of the number of children and older members on the increase in income.

The models are as follows:


(1) $$\begin{array}{l} y_{fz}=\theta_{1}+\gamma_{1}KIDs+\gamma_{2}OFFs+\gamma_{3}FARs+\gamma_{4i}CONs_{i}^{i=5}+\delta_{1i} \end{array}$$

(2) $$\begin{array}{l} y_{f}=\alpha_{1}+\beta_{1}KIDs+\beta_{2}OFFs+\beta_{3}FARs+\beta_{4i}CONs_{i}^{i=5}+\varepsilon_{1i} \end{array}$$

(3) $$\begin{array}{l} y_{yz}=\theta_{2}+\gamma_{5}OLDs+\gamma_{6}OFFs+\gamma_{7}FARs+\gamma_{8i}CONs_{i}^{i=5}\;+\delta_{2i} \end{array}$$

(4) $$\begin{array}{l} y_{y}=\alpha_{2}+\beta_{5}OLDs+\beta_{6}OFFs+\beta_{7}FARs+\beta_{8i}CONs_{i}^{i=5}+\varepsilon_{2i} \end{array}$$

*y*_*f*_ and *yfz*, *yy* and *yyz* denote the increase or decrease in household income after the shock of COVID-19 for poverty-stricken households and their counterparts and for households supporting children and those supporting older members, respectively. α_1_ and θ_1_, α_2_ and θ_2_ are constant terms. *KIDs*, *OLDs*, *OFFs* and *FARs* are the number of children, older members, and agricultural and nonagricultural laborers, respectively, in poverty-stricken households. $CONs_{i}^{i=5}$ is the *i*th control variable. β_1_, β_2_, β_3_, β_5_, β_6_, β_7_, β_4*i*_, β_8*i*_, γ_1_, γ_2_, γ_3_, γ_5_, γ_6_, γ_7_, γ_4*i*_, *and γ*_8*i*_ are estimated coefficients, and ε_1*i*_, δ_1*i*_, ε_2*i*_, δ_2*i*_ are random disturbance terms.

A multiple linear regression model is used to test the relationship between policy measures and the human capital of poverty-stricken households. The correlation between policy measures and the human capital of poverty-stricken households is tested by selecting dependent family members and laborers as the dependent variables and the policy support measures obtained by poverty-stricken households as the independent variables. The models are constructed as follows:


(5) $$\begin{array}{l} y_{i}^{i=5}=\theta_{0}+\mu_{i}POLs_{i}^{i=7}+\delta_{0} \end{array}$$

where $y_{i}^{i=5}$ represents the human capital of the rural household, $POLs_{i}^{i=7}$ represents the policy measure obtained by the rural household, μ_*i*_ represents the estimated coefficient, and θ_0_ and δ_0_ represent the constant and disturbance terms, respectively.

Stepwise regression is used to test the mediating and suppression effects of the various policies. In Model 1 and Model 3, with the “government subsidy” term in the control variables eliminated, the significance and size of the regression coefficients of the number of agricultural laborers and nonagricultural laborers in poverty-stricken households with older members and poverty-stricken households with children before and after the introduction of “government subsidies” are important to observe. An increase in the coefficient and its significance indicates that the policy measures have a mediating effect; otherwise, they have a suppressive effect. The models are as follows:


(6) $$\begin{array}{l} y_{fz}=\theta_{3}+\gamma_{9}KIDs+\gamma_{10}OFFs+\gamma_{11}FARs+\gamma_{12i}CONs_{i}^{i=4}+\delta_{1i} \end{array}$$

θ_3_ *are constant terms*, γ_9_, γ_10_, γ_11_, *and γ*_12*i*_ are estimated coefficients.


(7) $$\begin{array}{l} y_{yz}=\theta_{4}+\gamma_{13}OLDs+\gamma_{14}OFFs+\gamma_{15}FARs+\gamma_{16i}CONs_{i}^{i=4}\;+\delta_{2i} \end{array}$$

θ_4_ *are constant terms*, γ_13_, γ_14_, γ_15_ *and γ*_16*i*_ are estimated coefficients.

## 4 Results

### 4.1 Differences in the effects of human capital on income increases between poverty-stricken and nonpoverty-stricken households

As mentioned in Section 3.1.2, most households with dependency burdens were child-supporting and older-supporting households. To explore the effectiveness of TPA policy measures in a specific group accurately, child-supporting and older-supporting are selected as the research subjects. The dependent variables, core variables, and control variables in Table 2 are combined in Models 1–4 to obtain the relationships between human capital and income growth for poverty-stricken and nonpoverty-stricken households.

#### 4.1.1 Differences in income increases among households with different dependency burdens

As shown in Table 3, Models 1 and 3 and Models 2 and 4 show the results of the regressions of human capital and household income growth for poverty-stricken households and nonpoverty-stricken households, respectively.

**Table 3:** Differences in the increase in human capital income of poverty-stricken households.

**Variable**	**Households with children**		**Households with older members**	
	**Poverty-stricken households**	**Nonpoverty-stricken households**	**Poverty-stricken households**	**Nonpoverty-stricken households**
	**Model 1**	**Model 2**	**Model 3**	**Model 4**
Number of older members			0.0683^*^	0.0373
Number of children	0.1165^*^	−0.0242		
Number of people with disabilities	−0.0073	0.0031	−0.0427	0.01
Number of non-agricultural laborers	−0.1930^***^	0.0235	−0.1186^***^	−0.0925^***^
Number of agricultural laborers	0.062	−0.0163	0.0458^*^	0.0123
Household size	−0.0891^**^	0.1697^**^	−0.0167	−0.019
Hospitalization or not	0.0602	−0.1024	−0.0073	−0.1421^***^
Government subsidies	0.0243^**^	0.0894^*^	0.0603^***^	0.0551^***^
Total household income	0.1406	−0.4038^***^	−0.2209^***^	−0.0522^*^
Constant term	1.0068	5.5619^***^	4.4182^***^	2.9335^***^

^*^p < 0.1.

^**^p < 0.05.

^***^p < 0.01.

The regression results of Model 2 indicate that the number of children negatively affects the increase in income for nonpoverty-stricken households. Children are the main consumers in the family. They cannot increase household income and require adult laborers to care for them in daily life, which limits the output of adult laborers. Consequently, the number of children has a negative effect on the increase in income for child-supporting households. Rahman (70) and Zhang et al. (71) reported that the number of children can worsen the poverty status of poverty-stricken households and affect their quality of poverty alleviation. However, the regression results of Model 1 indicate that the number of children positively affects the increase in income for poverty-stricken households supported by policy measures, with a regression coefficient of 0.1165, which is statistically significant (*p* < 0.1, Table 3). The contradictory results between poverty-stricken households and nonpoverty-stricken households imply that TPA policy measures alleviate the dependency burden of children in poverty-stricken households.

Similarly, the regression results of Model 4 indicate that the number of older members positively affects the income increase for older-supporting households. In rural China, the income of older members is an important component of household income for poverty-stricken households. First, people older than 60 years can receive endowment insurance every month. Older members are the main participants in rural production activities, engaging in agricultural production and nearby work to earn some income (72). Therefore, the number of older members positively affects the increase in income in nonpoverty-stricken households. Additionally, the regression results of Model 3 indicate that the number of older members has a significantly positive effect on the income increase for poverty-stricken households benefitting from TPA policy measures (coefficients are 0.0683, and significance is *p* < 0.1; Table 3). The number of older members has a greater effect on the increase in income for poverty-stricken households than nonpoverty-stricken households. The results show that TPA policy measures have enhanced the positive impact of older members on the increase in income of poverty-stricken households.

#### 4.1.2 Difference in income increase by number of laborers in the household

The results of Models 1–4 indicate that the number of nonagricultural laborers negatively affects income increases for children-supporting poverty-stricken households, older-supporting poverty-stricken households, and older-supporting nonpoverty-stricken households, with coefficients of −0.1930, −0.1186, and −0.0925, respectively, at the *p* < 0.01 significance level (Table 3). COVID-19 affected the employment of nonagricultural laborers. After the outbreak of COVID-19 in February 2019, a shutdown policy was implemented throughout China. Many enterprises shut down or even went bankrupt due to the COVID-19 pandemic. Moreover, the employment period of nonagricultural laborers was shortened, and some lost employment (55, 56). However, in our estimation, the number of nonagricultural laborers has a nonsignificant effect on the increase in income for children-supporting nonpoverty-stricken households. One explanation may be that the main laborers in child-supporting households are usually young and have little work experience, which results in limited income. These households and their immediate family members may have good family conditions, enabling them to accumulate some assets. Upon becoming independent, the adults in these households can receive more help from their parents (47). Correspondingly, Model 2 shows that family size has a significant positive effect on income increases for general households, further explaining the supportive role of family members. Therefore, the number of nonagricultural laborers has a nonsignificant effect on the increase in income for children supporting nonpoverty-stricken households.

Similarly, the effect of the number of agricultural laborers on income increase is not significant for children-supporting poverty-stricken households, children-supporting nonpoverty-stricken households or older-supporting nonpoverty-stricken households. The reason for this may be that agricultural industrialization and production efficiency are low in the study area. Agricultural income represented 21.55% of the total income of the surveyed poverty-stricken households and 16.37% of the total income of the surveyed nonpoverty-stricken households. The pandemic had little impact on the agricultural production of poverty-stricken households, with 89.25% of the poverty-stricken households stating that COVID-19 had little or no impact on agricultural production. However, the effect of the number of agricultural laborers on income increase has a significant positive effect on older person-poverty-stricken households, with a significant regression coefficient of 0.0458 (*p* < 0.1, Table 3). This is because the older people are the main participants in rural production and life at present, and older person-poverty-stricken households receive more agricultural assistance from TPA (28). When the impact of the COVID-19 pandemic restricted the migration of nonagricultural laborers, nonagricultural laborers from poverty-stricken households began to engage in agricultural production. In this situation, the basic agricultural production provided by their older household members was able to support them. Moreover, the increase in the number of agricultural laborers further enhanced the efficiency of agricultural output, which increased income in older-supporting households.

### 4.2 The connection between TPA policy measures and poverty-stricken households' ability to cope with the COVID-19 shock

#### 4.2.1 Relationships between TPA policy measures and human capital in poverty-stricken households

Policy measures are positively connected with the human capital of poverty-stricken households (Table 4). With respect to safety net policies, subsistence allowances (Dibao) have a significantly positive effect on poverty-stricken households with a greater number of older and disabled members, indicating that safety net policies can effectively identify household members who need support and provide effective assistance. The renovation of rural dilapidated houses has no significant positive effect on the human capital of poverty-stricken households. This is because ensuring housing safety is an important standard for poverty-stricken households being lifted out of poverty. Whether poverty-stricken households can obtain assistance for home renovation depends on the quality of their housing, which has no direct correlation with their human capital.

**Table 4:** Relationship between policy measures and human capital of poverty-stricken households.

**Variable**		**Older member**	**Children**	**Disabled**	**Nonagricultural laborers**	**Agricultural laborers**
Safety nets	Subsistence allowances (SA)	0.0855^***^		0.2028^***^		
	Renovation of rural dilapidated houses (RH)				
Cargo nest	Public welfare jobs (WJ)		0.0637^***^			0.2500^***^
	Relocation (RL)		0.2209^***^		0.1876^***^	
	Industry share (IS)					0.1279^***^
	Land transfer (LT)			0.0085^**^		
	Microcredit (CR)		0.1378^***^		0.0994^***^	0.2849^***^

^*^p < 0.1.

^**^p < 0.05.

^***^p < 0.01.

In terms of cargo net policies, public welfare jobs and microcredits have a significant relationship with poverty-stricken households with a higher number of children and agricultural laborers. In fact, for poverty-stricken households, the income of nonagricultural laborers is not sufficient to support their dependents. Therefore, to resolve the conflicts between family livelihood and childcare, one of the parents tends to stay at home to farm. The government fully supports the value of agricultural laborers by providing public welfare jobs and microcredits, which enhances the efficiency of agricultural output and increases the income of poverty-stricken households.

Relocation has a significantly positive effect on households with more children and nonagricultural laborers. Relocation enables poverty-stricken households to voluntarily migrate from remote mountainous areas to areas with more extensive infrastructure. In the context of relocation, households with children are able to integrate into the new environment. In addition, poverty-stricken households with children tend to relocate to seek employment opportunities and a better education environment.

Poverty-stricken households can participate in the development of village collective industries through their labor and land sharing. Rural households with some labor capacity are usually unwilling to relinquish their own farmlands and participate in the village collective industry through labor sharing. Thus, industry share is positively associated with the number of agricultural laborers in poverty-stricken households. Similarly, disabled members without the ability to work participate in the development of village collective industries through land sharing. Hence, land transfer is significantly and positively correlated with the number of disabled members in poverty-stricken households.

#### 4.2.2 Mechanisms through which policy measures enhance poverty-stricken households' response to the COVID-19 shock

The role of poverty-stricken households' human capital in household income is examined by comparing the results before and after government subsidies are added to the multiple regression model, where government subsidies constitute the independent variable.

TPA policy measures provide effective support to dependent members in poverty-stricken households. As presented in the regression results of Model 6 and Model 1, Model 7 and Model 3, after adding government subsidies to the equation, the regression coefficient of the number of children in child-supporting households increases from 0.1158 to 0.1165, with no significant change. The regression coefficient of government subsidies reaches 0.0243 and is statistically significant (*p* < 0.05). Moreover, the regression coefficient of the number of older members for older-supporting households decreases from 0.0853 to 0.0683. The regression coefficient of government subsidies is significant, reaching 0.0603 (*p* < 0.01; Table 5). Policy measures are directly related to the number of household members needing support in poverty-stricken households. Policy measures provide support for these dependent household members by increasing household income, which reduces the economic burden of poverty-stricken households.

**Table 5:** Policy measures affecting the income increase of human capital in poverty-stricken households.

**Variable**	**Households with children**		**Households with older members**	
	**Model 6**	**Model 1**	**Model 7**	**Model 3**
Number of older members			0.0853^**^	0.0683^*^
Number of children	0.1158^*^	0.1165^*^		
Number of people with disabilities	0.0029	−0.0073	−0.0355	−0.0427
Number of non-agricultural laborers	−0.2132^***^	−0.1930^***^	−0.1402^***^	−0.1186^***^
Number of agricultural laborers	0.0598	0.0620	0.0373	0.0458^*^
Household size	−0.0794^*^	−0.0891^**^	−0.0194	−0.0167
Hospitalization or not	0.0705	0.0602	−0.0092	−0.0073
Total household income	0.1337	0.1406	−0.2148^***^	−0.2209^***^
Government subsidies		0.0243^**^		0.0603^***^
Constant term	1.2197	1.0068	4.8694^***^	4.4182^***^

^*^p < 0.1.

^**^p < 0.05.

^***^p < 0.01.

Policy measures can mitigate the impact of COVID-19 on nonagricultural laborers and enhance the output benefits of agricultural laborers. As shown in Model 6 and Model 1, Model 7 and Model 3, after adding government subsidies to the equation, the negative impact of the number of nonagricultural laborers decreases from 0.2132 to 0.1930. The positive impact of the number of agricultural laborers increases from 0.0598 to 0.0620 but is not significant. Similarly, the negative impact of the number of nonagricultural laborers for older-supporting households decreases from 0.1402 to 0.1186. Similarly, the positive impact of the number of agricultural laborers increases from 0.0373 to 0.0458 and is statistically significant for older-supporting households (Table 5). Hence, TPA policy measures can improve the ability of poverty-stricken laborers to cope with COVID-19 shocks by mitigating the adverse effects of COVID-19 on income for nonagricultural laborers and increasing the income of agricultural laborers.

## 5 Discussion

Scholars have noted that local governments play an important role when poverty-stricken households are exposed to severe negative external shocks (73). Poverty-stricken households in developing countries face shocks that undermine their wellbeing, and policy measures, such as safety net and cargo net policies, prevent poor populations from falling into chronic poverty (74). While there is wide heterogeneity in the coverage and support of policy measures across countries (75), such policies can contribute substantially to the livelihood of beneficiaries (76). A study by Janvry et al. (77) showed that safety nets can enable households to make better investments in their future—both in the human capital of their children and in the livelihoods of income earners. Hansen et al. (78) discussed the roles that climate-risk management interventions can play in efforts to reduce rural poverty and the need for further research on identifying and targeting environments (e.g., soil and climate) and farming populations (e.g., labor endowments) where improved climate risk management could accelerate efforts to reduce rural poverty. However, the above studies do not discuss the mechanism through which policy measures enhance poverty-stricken households' ability to cope with external shocks in terms of human capital. Therefore, this paper goes beyond merely verifying the role of policy measures in the response of poverty-stricken households to COVID-19 to analyze in depth the mechanism through which policy measures influence this response.

According to our results, first, poverty-stricken households' access to policy measures is closely related to their human capital endowments, as mentioned in Section 4.2.1. Policy measures such as safety nets and cargo nets are implemented to provide a protective barrier for household livelihoods and prevent negative effects from the COVID-19 shock. Subsistence allowances (Dibao), such as safety nets, are positively connected with the number of older and disabled members in poverty-stricken households. In fact, older people have become the main force of agricultural production for poverty-stricken households in rural China. However, the effect of COVID-19 significantly decreases the likelihood of older members participating in agricultural labor and reduces their agricultural work time (79), affecting rural household income. In this situation, safety net policies can be used to identify household members who need support and provide effective assistance. Moreover, cargo net policies are positively associated with the number of agricultural or nonagricultural laborers in a household and can ensure an increase in income for poverty-stricken households. Specifically, public welfare jobs, microcredits and industry shares are positively connected with the number of agricultural laborers in poverty-stricken households. Although nonagricultural laborers must return to their hometowns because external shocks affect family members, they can still maintain stable household income.

Second, policy measures have buffered some of the negative impacts of COVID-19 on poverty-stricken households and reduced the likelihood of household exposure to external shocks through the impact on households' human capital. Unlike this paper, Li et al. (3) used the health level and education level of the labor force as an indicator to measure the human capital endowment and showed that policy measures did not effectively improve the human capital of poverty-stricken households in the short term. This paper considers how policy measures affect the ability of poverty-stricken households to cope with external shocks, by adopting household population composition as human capital. Safety net policies ensure that poverty-stricken households that suffer from external shocks obtain stable income and relieve the stress caused by dependent members in the household. Similarly, Mnyanga et al. (80) reported that households benefiting from various safety net programs during the COVID-19 pandemic were less likely to reduce food consumption and rely on savings. Safety net policies are likely to be beneficial, but their impact is sometimes limited (73). Therefore, it is necessary to consider the effectiveness of cargo net policies, which enhance the economic performance of dependent family members and agricultural laborers in poverty-stricken households suffering from external shocks. A study by Mahmud et al. (81) revealed that microcredits had a positive effect on increasing risk management capacity and supporting household income for fish farmers. In addition, land transfer (82), relocation (83), and industry share (84) have been proven to effectively increase the income of rural households. In China's TPA policy measures, the synergies between safety and cargo nets enhance the stability of poverty-stricken households and support their long-term ability to cope with external shocks through human capital.

**Figure 3:**
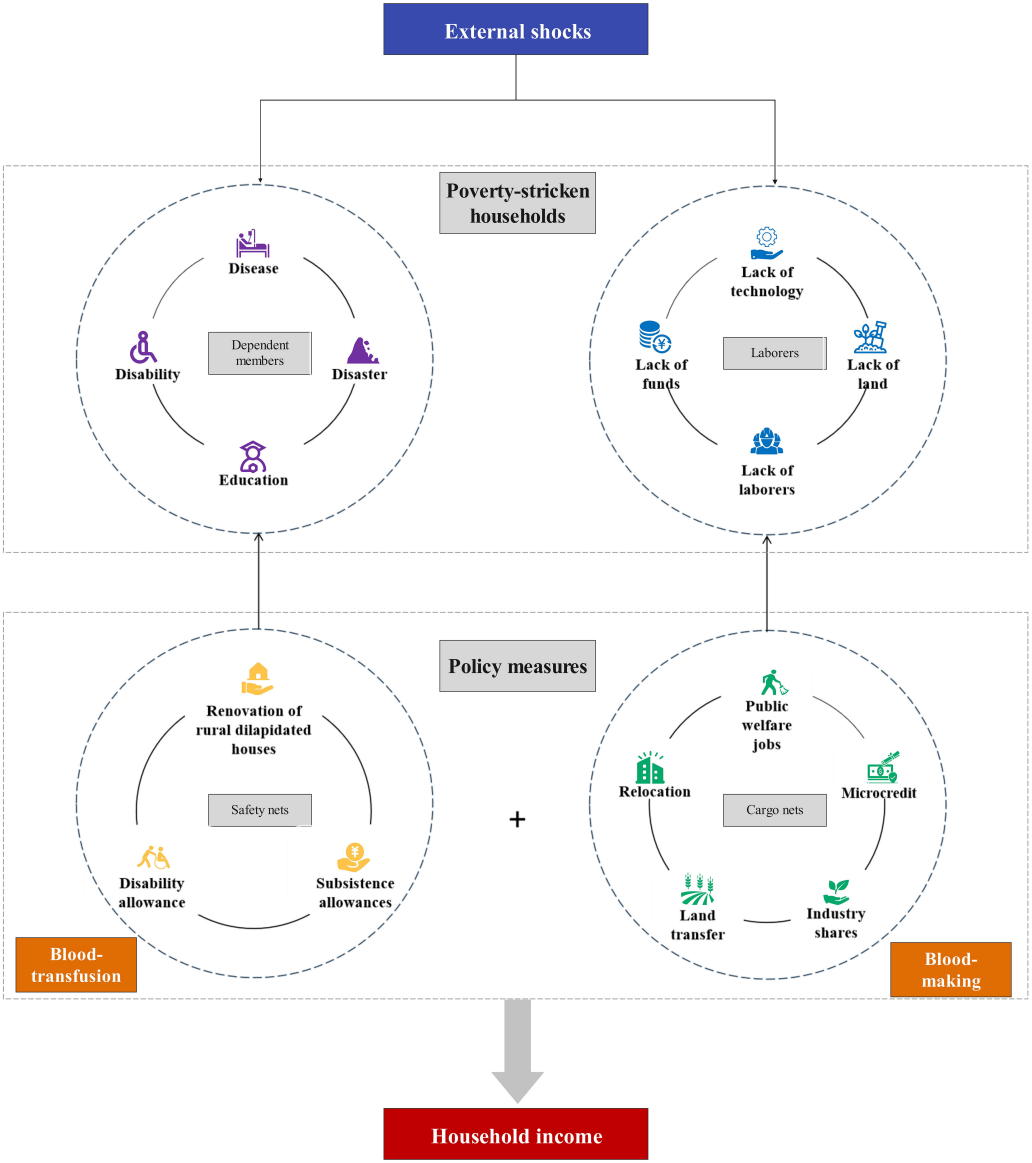
The role of policy measures in influencing the human capital of poverty-stricken households.

In conclusion, successful protection against the negative effects of COVID-19 has been achieved in China because the household human capital endowment of poverty-stricken households is connected with governmental policy measures. The role of policy measures in improving the coping ability of these poverty-stricken households in terms of human capital can be summarized in two aspects (Figure 3). On the one hand, safety net policy measures guarantee support for dependent household members, thereby buffering the adverse effects of the number of dependent household members on income for poverty-stricken households. On the other hand, cargo net policy measures alleviate the adverse effects of external risks on the income of nonagricultural laborers, enhance the efficiency of agricultural output and increase household income. Through these two types of policies, the impact of short-term risk on income for poverty-stricken households can be effectively mitigated, thereby preventing poverty-stricken households from falling back into the poverty trap.

## 6 Conclusions and policy recommendations

Given that increased external shocks severely affect the livelihoods of poverty-stricken households, understanding how poverty-stricken households cope with external shocks under policy measures is highly practical. However, studies on how policy measures affect the ability of poverty-stricken households to cope with external shocks remain relatively limited. Thus, this paper aims to fill the existing knowledge gap by empirically examining policy measures and exploring how the ability of poverty-stricken households to cope with external shocks is enhanced with the help of support policies and their underlying mechanisms. This paper empirically how policy measures enhance the ability of poverty-stricken households to use their human capital to cope with external shocks. The data utilized in this paper were obtained from a questionnaire survey of 6,463 poverty-stricken households in southwest China. Our results contribute to the discussion on the role of policy measures in providing support for dependent household members and household laborers. The following conclusions are drawn:

1) Policy measures can alleviate the adverse income effects associated with dependent household members for poverty-stricken households. Policy measures can reverse the negative effects of children and amplify the positive effects of older members on poverty-stricken households' income.
2) Poverty-stricken households' access to support policies is closely related to their human capital endowments. Safety net policies are closely related to household dependent members, whereas cargo net policies are closely related to the composition of poverty-stricken household laborers. The ability of poverty-stricken household capital to cope with external shocks is guaranteed by these two types of policies.
3) Policy measures can alleviate the adverse effects of the COVID-19 shock on the increase of nonagricultural laborers and enhance the income of agricultural laborers. In China, the government has vigorously developed industries and improved infrastructure in rural areas, which has increased the industrial efficiency of agriculture. During the COVID-19 outbreak, nonagricultural laborers had to return home, thereby increasing the number of agricultural laborers. The addition of agricultural laborers improved the output efficiency of agricultural production, thereby reducing the impact of COVID-19 on nonagricultural laborers.

To address related challenges, some policy recommendations can be proposed for enhancing poverty-stricken households' income stability and ability to cope with external shocks through human capital. First, revitalizing rural industries is important for enhancing households' ability to cope with external shocks. This enhances the efficiency of agricultural output, ensuring that poverty-stricken households acquire a certain level of agricultural income when household laborers are impacted by external risk, thereby mitigating the impact of external risk on household income. Additionally, connecting the development of rural industries with household income is vital. In the process of developing rural industries, it is important to focus on revitalizing the livelihood assets of households, linking industry development with household income and enhancing household assets. Finally, the labor resources of those who stay in rural areas should be rationally utilized for agricultural work. It is particularly important to make rational use of rural labor resources by developing the agricultural industry, providing agricultural technology training, and cultivating entrepreneurial leaders for wealth creation. Therefore, the agricultural output efficiency of agricultural laborers can be improved, and agricultural laborers can play a role in revitalizing rural industries.

Based on existing poverty research paradigms, this paper verifies the role of policy measures in the response of poverty-stricken households to COVID-19 shocks and further analyses in-depth how policy measures support poverty-stricken households' response. It is highly important to consolidate the achievements of poverty alleviation and achieve common prosperity. Therefore, the key to current research is enhancing the ability of poverty-stricken households to cope with external shocks and constructing monitoring and assistance mechanisms to prevent them from returning to poverty. Finally, a limitation of this study is that our data on the change in rural household income are obtained based on the subjective evaluation of poverty-stricken households, which may introduce a certain degree of error. Future studies may apply quantitative analysis to multiperiod data from poverty-stricken households to accurately measure the increase or decrease in income and thereby reduce the interference of rural households' subjective judgments on the research results.

## Data Availability

The original contributions presented in the study are included in the article/supplementary material, further inquiries can be directed to the corresponding author.
